# On the Use of Bofareira (“Ricinus Communis” of Botanists) as a Means Adopted by the Natives of the Cape De Verd Islands to Excite Lactation

**Published:** 1850-12

**Authors:** J. O. M’William

**Affiliations:** Surgeon to the Honorable the Board of Customs


*On the use of Bofareira ("Ricinus Communis" of Botanists) as a means adopted by the natives of the Cape De Verd Islands to excite Lactation. By* J. O. M'William, M. D., F. R. S., R. N., Surgeon to the Honourable the Board of Customs.

While engaged in an official investigation into the nature and history of a yellow fever epidemy, prevailing in the Island of Boa Vista, in the Cape de Verds, during the year 1846, my attention was called to a remedy commonly had recourse to there, and in the other islands of the group, to accelerate and increase the flow of milk from the breasts of child-bearing women, in cases where that secretion was tardy in appearing, or deficient in quantity when it did appear.

I also learnt that on occasions of emergency, this remedy could be successfully applied to a still more important use, namely, to produce milk in the breasts of women who are not childbearing, or who even have not given birth to, or suckled, a child for many years.

The leaves of a plant, called in the language of the country, Bofareira, but which, in reality, is the "Ricinus communis" of botanists, and, occasionally, the leaves of the "Jathropha curcas," both belonging to the natural family *euphorbiaciæ*, are the means by which these interesting if not extraordinary results are produced.

The Bofareira grows in most, if not all, the Cape de Verd Islands. That used by the natives for the purposes I have mentioned is called by them *white* bofareira, to distinguish it from what appears to be nothing more than a variety of the same species, the *red* bofareira. The *white* or that which possesses galactagogue qualities, is recognized by the natives by the light green colour of the stem of the leaf, whilst the leaf stem of the *red* is of a purplish red hue The latter plant is carefully avoided, as it it is said to be a powerful irritant, and, if applied, as it occasionally has been, by mistake for the *white*, it produces an immediate and often immoderate flow of the menses.

In cases of childbirth, when the appearance of the milk is delayed (a circumstance of not unfrequent occurrence in those islands,) a decoction is made by boiling well a handful of the *white* Bofareira in six or eight pints of spring water. The breasts are bathed with this decoction for fifteen or twenty minutes. Part of the boiled leaves are then thinly spread over the breasts, and allowed to remain until all moisture has been removed from them by evaporation, and probably in some measure by absorption. This operation of fomenting with the decoction and applying the leaves, is repeated at short intervals until the milk flows upon suction by the child which it usually does in the course of a few hours.

On occasions where milk is required to be produced in the breasts of women who have not given birth to, or suckled, a child for years, the mode of treatment adopted is as follows:—

Two or three handfuls of the leaves of the Ricinus are taken and treated as before. The decoction is poured, while yet boiling, into a large vessel, over which the woman sits so as to receive the vapour over her thighs and generative organs, cloths being tucked around her so as to prevent the escape of the steam. In this position, she remains ten or twelve minutes, or until the decoction cooling a little, she is enabled to bathe the parts with it, which she does for fifteen or twenty minutes more. The breasts are then similarly bathed, and gently rubbed with the hands; and the leaves are afterwards applied to them in the manner already described. These several operations are repeated three times during the first day. On the second day, the woman has her breasts bathed, the leaves applied, and the rubbing repeated three or four times. On the third day, the sitting over the steam, the rubbing, and the application of the leaves to, with the fomentation of, the breasts are again had recourse to. A child is now put to the nipple, and, in the majority of instances, it finds an abundant supply of milk.

In the event of milk not being secreted on the third day, the same treatment is continued for another day, and if then there still be a want of success, the case is abandoned, as the person is supposed not to be susceptable to the influence of the Bofareira.

Women with well-developed breasts are most easily affected by the Bofareira. When the breasts are small and shrivelled, the plant then is said to act more on the uterine system, bringing on the menses, if their period be distant, or causing their immoderate flow if their advent be near.

Exposure to cold is carefully avoided by persons who are being brought under the influence of the Bofareira. These scrupulously abstain from wetting with cold water either the hands or the feet.

Maria, a dark mulatto women, with wooly hair, thirty years of age, tall, stout, and well-formed; menstruating regularly; the mother of three children, the youngest of whom was three years old and had been weaned when under the age of one year, was brought before me by Dr. Almeida, of Boa Vista, on the morning of the 30th of June, 1846, for the purpose of being submitted to the action of the Bofareira. She stated, that when her child was weaned, every trace of milk disappear- from her breasts in the course of a few days. I could not detect any signs of pregnancy. The breasts were like those of negro women in general who have borne children, pendulous and flabby. No sign of milk was given out from them upon careful examination of the nipple.

The baths, fomentations, the application of the leaves, friction, suction, &c., were adopted in the manner and order I have already described. On the second day there was a slight oozing of a serous-looking milk from the nipples, with slight increase of size in the areolar portion of the breast. On the third day, the milk increased in quantity, and less watery. On the morning of the fourth day there was an evident enlargement of the lower part of the mamma, and milk flowed abundantly upon the application of a child to the nipple.

The use of the Bofareira in cases of childbirth, to accelerate the flow of milk, is common, but comparatively rare as the means of procuring a wet-nurse. Some instances of the latter kind occurred, in consequence of the death of mothers with children at the breast during the progress of the Boa Vista epidemy of 1845-46, which decimated a population consisting almost wholly of blacks, with a few Europeans—Portugese and English—and a small proportion of mixed negro and European blood.

Generally, however, this use of the Bofareira is seldom called for. Death in childbirth, or prolonged illness after parturition, sometimes requires a kind relative or charitable neighbor, who, for the safety of the offspring, places herself under the influence of the bofareira.

The son of a wealthy landed proprietor of San Nicolao, (well known to my friend, Mr. George Miller, of that Island,) a remarkably hale and robust-looking man, was wet-nursed by a woman who gave him milk produced by the Bofareira. The nurse in this instance had borne two children in early life. Her husband died shortly after the birth of her second child ; she lived in a state of virtuous widowhood, and it was *many* years after the death of her husband that she so generously submitted herself to the Bofareira, and nursed the infant in question.

Consul-General Rendall, of the Cape de Verds, informs me that a lady, a native of Boa Vista, now residing at San Antonio, and the wife of one of the foreign consuls, had a daughter in 1843. "Having very little milk," says Mr. Consul Rendall, "she caused an old female servant to be prepared with the bofareira, and to act as wet-nurse, which she did in the most satisfactory manner, having plenty of good milk, although she had not had a child for ten years previously. The child is now (March, 1847) a healthy one, and well grown. In short," continues Mr. Randall, "women who use the Bofareira are in two or three days in order sufficient to nurse the child of a queen."

I have not been able to ascertain, from personal observation, or from any very accurate information, what effect the Bofareira has upon virgins, or upon those who, although they have not borne children, are nevertheless not virgins. As regards the latter class, however, an intelligent native midwife assured my most able and observant friend, Mr. George Miller, of San Nicolao, that the effect of the administration of the Bofareira is much the same upon them as upon child-bearing women.

In some cases, but rarely, the decoction of the Bofareira is taken internally, with the view of assisting the action of its external application.

I regret not having been informed of the alleged difference in the action of the white and red Bofareiras, while I was at the Cape de Verds, that I might have examined the latter plant upon the spot.

The seeds of each plant were, however, kindly forwarded to me by Mr. George Miller, and Sir William Hooker most readily and obligingly examined them. Sir William, in a note to me, says, "What you mark as red Bofareira, and as white Bofareira, are both, not only of the genus 'Ricinus,' but also of one and the same species—viz., Ricinus communis, the common palma Christi, or castor-oil plant. In our gardens, as well as abroad, the plants vary, and your two plants vary a little in the form and size of the seed, and especially in the color, but they are one and the same species."

It is thus evident that the white and red Bofareiras, if they differ at all, can only be varieties of the same species. It is known, however, that certain varieties of other plants, as thyme, mint, &c., do yield different properties, and such may be the case with the Bofareiras.

I have thus stated all the facts that have come to my knowledge regarding this galactagogue of the Cape de Verds, which I consider to be well worthy of a fair trial in this country. Should its action in our more temperate regions be similar to that which it exerts within the tropics, an interesting field of inquiry will be opened, as regards its hygienic, medicinal, medico-legal, and other relations.

These, however, are points, the consideration of which had better be reserved until it has been determined, by experiment, how far the Bofareira can be successfully introduced into the practice of this country.

[*Note.*—Dr. Tyler Smith, to whom I showed my paper before my visit to Edinburgh, has written to inform me that he has in several cases tried the Bofareira in the manner described by me; and he assures me that the effects of the plant grown in this country fully bear out the facts I have detailed respecting the use of this plant in the Cape de Verd Islands.]—*London Lancet.*

